# Context-dependent immunomodulation by caspofungin: inflammatory attenuation involving spleen tyrosine kinase signaling in monocytic-cell systems versus pathogen-mediated immune amplification

**DOI:** 10.1093/femsml/uqag028

**Published:** 2026-08-05

**Authors:** Kazuhiro Itoh, Hiromichi Iwasaki, Masamichi Ikawa, Yasuhiko Mitsuke

**Affiliations:** Department of Community Health Science, Faculty of Medical Sciences, University of Fukui, Fukui 910-1193, Japan; Department of Community Health Science, Faculty of Medical Sciences, University of Fukui, Fukui 910-1193, Japan; Department of Community Health Science, Faculty of Medical Sciences, University of Fukui, Fukui 910-1193, Japan; Department of Internal Medicine, NHO Awara National Hospital, Awara, Fukui 910-4272, Japan

**Keywords:** caspofungin, echinocandin, immunomodulation, spleen tyrosine kinase, β-glucan, *Candida*

We read with great interest the study by Shehata et al. (2026), which provides an important transcriptomic view of how caspofungin shapes host antifungal immunity in a human whole-blood infection model. By comparing an echinocandin-susceptible *FKS* wild-type *Candida albicans* strain with an echinocandin-resistant strain carrying an *FKS1* S645P mutation, the authors showed that caspofungin at 2 μg/ml not only reduced fungal viability in the susceptible strain but also amplified host immune transcriptional programs. In contrast, caspofungin induced only minimal host transcriptional changes in uninfected blood or during infection with the resistant strain. These findings indicate that immunomodulation in whole blood is largely mediated through drug–pathogen interactions rather than baseline stimulation of host leukocytes.

This conclusion invites reconsideration of earlier observations from simplified immune-cell systems. In our previous study, caspofungin suppressed zymosan-induced production of inflammatory cytokines and chemokines in THP-1 monocytic cells, including tumor necrosis factor-α (TNF-α), interleukin (IL)-1β, IL-6, IL-8, interferon-γ-inducible protein 10 (IP-10), monocyte chemoattractant protein-1 (MCP-1), and macrophage inflammatory protein-1α/β (MIP-1α/β) (Itoh et al. 2021). At 30 μg/ml, caspofungin reduced activation of spleen tyrosine kinase (Syk) and downstream molecules, including inhibitor of nuclear factor-κBα (IκBα), mitogen-activated protein kinases (MAPKs), nuclear factor of activated T cells (NFAT), and caspase-1. Pharmacological Syk inhibition reproduced the reduction in TNF-α release. Importantly, these effects were concentration dependent: cytokine suppression was most evident at 30 μg/ml, whereas 1 μg/ml did not reduce cytokine levels. Although direct comparison is limited by different models and readouts, this concentration dependence is compatible with the minimal host response to 2 μg/ml caspofungin in uninfected whole blood reported by Shehata et al. (2026).

A useful framework is to distinguish two mechanistically different layers of caspofungin-associated immunomodulation. The first is inflammatory attenuation involving Syk signaling in a ligand-stimulated monocytic-cell system. THP-1 cells are a leukemia-derived transformed cell line and do not fully reproduce primary circulating monocytes. In contrast, whole blood preserves primary monocytes, transcriptionally active neutrophils, lymphocytes, platelets, plasma proteins, complement, and intercellular communication. Neutrophils constitute a major leukocyte population in blood and can shape monocyte responses through phagocytosis, oxidative burst, degranulation, chemokine release, and cellular crosstalk. Moreover, zymosan is a relatively static particulate stimulus, whereas viable *Candida* undergoes dynamic cell-wall remodeling, morphogenetic transition, complement opsonization, and engagement of C-type lectin receptors, complement receptor 3, and Fcγ receptors. Although these pathways may converge on Syk, their magnitude and kinetics differ substantially between purified-ligand stimulation and whole-blood infection.

The second layer is pathogen-mediated immune amplification when caspofungin acts on viable, susceptible fungi. Inhibition of β-1,3-glucan synthesis perturbs fungal cell-wall architecture and may expose immunostimulatory β-glucan, enhancing pattern-recognition receptor engagement. In whole blood, this remodeling can coordinate innate recognition, phagosome maturation, interferon-associated pathways, antigen presentation, and adaptive immune polarization. The resistant *FKS1* mutant included by Shehata et al. already provides important support for this model: when fungal susceptibility and drug-induced remodeling were absent, caspofungin produced little additional transcriptional response. Thus, effective fungal targeting appears necessary to generate the dominant immune-amplifying signal.

Pharmacological context may further explain why Syk-associated attenuation was not evident in whole blood. Caspofungin is ∼97% plasma protein bound, so the unbound fraction available to influence host-cell signaling is expected to be lower in albumin-rich whole blood than in conventional culture medium containing fetal bovine serum (Stone et al. 2004). Host-cell-level attenuation may therefore require higher total and unbound exposure, together with robust ligand-induced Syk activation. The choice of readout is also important. Bulk transcriptomics is well suited to detecting coordinated pathogen-mediated immune amplification, but rapid post-translational changes in Syk signaling may occur without prominent changes in gene expression. Such effects may instead be detected through cytokine or chemokine release, leukocyte migration, phagocytosis, oxidative burst, or neutrophil degranulation. Consequently, minimal differential gene expression does not exclude non-transcriptional immunomodulatory effects.

This context-dependent model may also reconcile findings across fungal species and experimental platforms. Studies in THP-1 cells and echinocandin-treated *Candida* or *Aspergillus* indicate that immune consequences depend on drug concentration and unbound exposure, fungal susceptibility and morphology, cell-wall remodeling, immune-cell composition, complement activity, timing, and the selected biological readout (Hohl et al. 2008, Wheeler et al. 2008, Walker and Munro 2020, Henry et al. 2022).

A key experiment would cross the two models directly. Whole blood could be stimulated with zymosan, heat-killed *Candida*, or a genetically defined *FKS* mutant in the presence of a range of caspofungin concentrations, under conditions in which drug-induced fungal remodeling is minimal or absent. Demonstration of reduced Syk phosphorylation or altered leukocyte functions in these conditions would support a host-cell-level signaling phenotype in whole blood. Parallel comparison with susceptible viable *Candida* would determine when pathogen-mediated amplification predominates. Priority measurements should include total and unbound caspofungin, β-glucan exposure, Syk phosphorylation, cytokine and chemokine release, migration, phagocytosis, oxidative burst, and degranulation.

Caspofungin should therefore not be classified simply as immunostimulatory or immunosuppressive. Inflammatory attenuation involving Syk signaling in ligand-stimulated monocytic-cell systems may coexist with pathogen-mediated amplification of antifungal immunity after remodeling of susceptible fungal cell walls. This formulation does not imply direct biochemical inhibition of Syk by caspofungin; rather, it highlights how drug exposure, fungal susceptibility, cellular context, and analytical readout determine the observed immune phenotype. Recognizing this duality may broaden our understanding of antifungal efficacy as an integrated drug–host–pathogen interaction.

## Supplementary Material

uqag028_Supplemental_File

## Data Availability

No new data were generated or analyzed in support of this commentary.
