# Supplementary material for: Context-dependent immunomodulation by caspofungin: inflammatory attenuation involving spleen tyrosine kinase signaling in monocytic-cell systems versus pathogen-mediated immune amplification
Source: Microlife. 2026 Aug 5;7:uqag028. doi: 10.1093/femsml/uqag028 (PMC13479921; doi:10.1093/femsml/uqag028)
Supplement: Supplementary file 1 [file uqag028_supplemental_file.pdf]

Forte Science Communications

Forte, Inc., KDX Shinjuku 286 Bldg. 5F, Shinjuku 2-8-6, Shinjuku-ku, Tokyo 160-0022, Japan
Tel: +81-3-3353-3545 Fax: +81-3-3354-3845 Email: info@forte-science.co.jp

FORTE

June 5, 2026

To Whom It May Concern:

This is to confirm that Dr. Ito’s manuscript "Context-dependent immunomodulation by caspofungin: inflammatory attenuation involving spleen tyrosine kinase signaling in monocytic-cell systems versus pathogen-mediated immune amplification" has been edited by a native English speaker at Forte, Inc. Forte is a Japan-based company that employs editors and rewriters with a science background. We have been helping the Japanese scientific community to publish in international journals since 1987.

While this certificate confirms the authors have used Forte’s editing services, we cannot guarantee that additional changes have not been made after our edits. Should you require any additional information about our services, please do not hesitate to contact our office.

Sincerely,

Forte, Inc.

株式会社フォルテ之印

KDX Shinjuku 286 Bldg. 5F
2-8-6 Shinjuku, Shinjuku-ku
Tokyo 160-0022, Japan
Tel: +81-3-3353-3545
Fax: +81-3-3354-3845
Email: info@forte-science.co.jp
